# Flagellum-driven motility enhances *Pseudomonas aeruginosa* biofilm formation by altering cell orientation

**DOI:** 10.1128/aem.00821-25

**Published:** 2025-07-03

**Authors:** Guanju Wei, Jessica-Jae S. Palalay, Joseph E. Sanfilippo, Judy Q. Yang

**Affiliations:** 1 Saint Anthony Falls Laboratory, University of Minnesota 5635, Minneapolis, Minnesota, USA; 2 Department of Civil, Environmental, and Geo-Engineering, University of Minnesota 5635, Minneapolis, Minnesota, USA; 3 Department of Biochemistry, University of Illinois at Urbana-Champaign 14589, Urbana, Illinois, USA; Indiana University Bloomington, Bloomington, Indiana, USA

**Keywords:** biofilms, cell motility, fluid flow, shear, *Pseudomonas aeruginosa*

## Abstract

Bacterial motility plays a crucial role in biofilm development, yet the underlying mechanism remains not fully understood. Here, we demonstrate that the flagellum-driven motility of *Pseudomonas aeruginosa* enhances biofilm formation by altering the orientation of bacterial cells, an effect controlled by shear stress rather than shear rate. By tracking wild-type *P. aeruginosa* and its non-motile mutants in a microfluidic channel, we demonstrate that while non-motile cells align with the flow, many motile cells can orient toward the channel sidewalls, enhancing cell surface attachment and increasing biofilm cell density by up to 10-fold. Experiments with varying fluid viscosities further demonstrate that bacterial swimming speed decreases with increasing fluid viscosity, and the cell orientation scales with the shear stress rather than shear rate. Our results provide a quantitative framework to predict the role of motility in the orientation and biofilm development under different flow conditions and viscosities.

**IMPORTANCE:**

Biofilms are ubiquitous in rivers, water pipes, and medical devices, impacting the environment and human health. While bacterial motility plays a crucial role in biofilm development, a mechanistic understanding remains limited, hindering our ability to predict and control biofilms. Here, we reveal how the motility of *Pseudomonas aeruginosa*, a common pathogen, influences biofilm formation through systematically controlled microfluidic experiments with confocal and high-speed microscopy. We demonstrate that the orientation of bacterial cells is controlled by shear stress. While non-motile cells primarily align with the flow, many motile cells overcome the fluid shear forces and reorient toward the channel sidewalls, increasing biofilm cell density by up to 10-fold. Our findings provide insights into how bacterial transition from free-swimming to surface-attached states under varying flow conditions, emphasizing the role of cell orientation in biofilm establishment. These results enhance our understanding of bacterial behavior in flow environments, informing strategies for biofilm management and control.

## INTRODUCTION

Biofilms, characterized by aggregations of bacterial cells encased within exopolymeric substances, are widespread in both natural and engineered environments, including streams and rivers (1–3), maritime systems (4), water infrastructure (5, 6), and within the human body (7). Depending on the context, biofilms can either be detrimental or beneficial. For instance, they contribute to the fouling of medical equipment (8–10), promote chronic infections (7), and enhance bacterial resistance to antibiotics (11). On the other hand, biofilms can be beneficial as they have been used to increase the degradation of aromatic hydrocarbons (12), improve oil recovery efficiency (13), and remove wastewater contaminants (14). A thorough understanding of the factors that govern biofilm development is essential for predicting and managing biofilm behavior. While extensive research has been conducted on the molecular and genetic mechanisms of biofilms (15–17), there remains a gap in understanding how physical parameters, particularly bacterial swimming motility, contribute to the initiation of biofilm formation. In particular, how motility influences initial surface attachment and subsequent biofilm development, and how this process is modulated by fluid flow, remains poorly understood.

Surface-attached biofilm formation typically begins with the attachment of planktonic cells, a process regulated by cell swimming and surface motility (18–21). The formation of *Pseudomonas aeruginosa* biofilms has been extensively studied due to its significance as a common human pathogen and ability to form biofilms (22–27). *P. aeruginosa* cells possess a single polar flagellum, which enables swimming motility, and type IV pili, which facilitate surface attachment and twitching motility. Together, these structures allow *P. aeruginosa* cells to navigate toward surfaces and initiate biofilm formation (22–24, 28). While biofilm maturation is supported by the production of exopolymeric substances in response to environmental signals, recent studies show that biofilm development and attachment are also regulated by fluid flow and bacterial motility (22, 23, 29). Flagellar motility allows cells to explore surfaces, while pilus-driven twitching enhances irreversible attachment, influencing early biofilm development. For instance, Secchi et al. (29) reported that motile cells have a higher attachment density compared to non-motile cells, suggesting that cell motility is advantageous for the attachment of cells to surfaces, the initial stages of biofilm formation.

In addition to facilitating surface attachment, bacterial motility contributes to subsequent stages of biofilm development. O’Toole and Kolter (22) demonstrated that both flagellar and twitching motility are necessary for *P. aeruginosa* to form stable biofilms on abiotic surfaces. Specifically, they showed that mutants lacking flagella or type IV pili had significantly reduced biofilm formation due to impaired surface attachment. Similarly, Khong et al. (30) found that *P. aeruginosa* cells adjust their swimming speed and orientation when near surfaces, promoting stronger initial adhesion and enhancing early biofilm volume compared to non-motile mutants. Additionally, Siryaporn et al. (23) observed that *P. aeruginosa* could move upstream against fluid flow by using type IV pili, providing an advantage in colonizing surfaces. These findings underscore the need to consider cell motility in biofilm growth models, as most models mainly focus predominantly on nutrient concentration and diffusion and do not account for parameters related to bacterial motility (31, 32), even though several recent theoretical and experimental studies have begun to incorporate bacterial motility into biofilm modeling (27, 28, 33–37).

Similar to cell motility, fluid flow is known to regulate bacterial surface attachment and biofilm formation (25, 38–44). A recent study shows that the attachment rate of *P. aeruginosa* on curved surfaces increases with a decrease in flow velocity (29). Another study found that early-stage biofilm growth of *Pseudomonas putida* is inhibited when shear stress exceeds a critical threshold and that high-frequency flow fluctuations further suppress biofilm development (38, 39). These studies suggest that high flow can inhibit bacterial surface attachment, potentially reducing the subsequent biofilm formation. Consistent with this, our recent study demonstrates that biofilms cease to develop when the local flow velocity is greater than the bacterial swimming speed (38). To predict and control cell attachment and biofilm formation, it is essential to consider both the flow dynamics and bacterial cell motility. Consistently, Secchi et al. (29) showed that cell motility can impact bacterial surface attachment efficiency, and the degree of such impacts varies with fluid flow velocity. They developed a mathematical model to predict the cell attachment efficiency based on both fluid flow and bacterial swimming speed. Further study is needed to understand the influence of the interplay between flow and cell motility on biofilm formation; however, such a study is currently lacking due to a lack of biofilm-development experiments with controlled flow conditions and bacterial motility.

In this study, we investigate how the interactions between swimming behavior and fluid flow impact the trajectories of *Pseudomonas aeruginosa* cells and the subsequent development of biofilms. *P. aeruginosa* is a versatile pathogen that can form biofilms and impact a broad spectrum of hosts (45–47). We directly visualized the cell trajectories and the orientation of cells using a high-speed camera and quantified the biofilm growth over time using confocal laser scanning microscopy. By comparing the trajectories and biofilms of motile *P. aeruginosa* cells with those of non-motile mutants, we demonstrate the role of bacterial cell motility in cell attachment and biofilm formation under various flow conditions. Furthermore, repeating the experiments under various fluid viscosities, we examine whether shear rate or shear stress controls cell orientation and subsequent biofilm formation.

## RESULTS

### Flagellum-driven cell motility enhances biofilm formation

**Fig 1:**
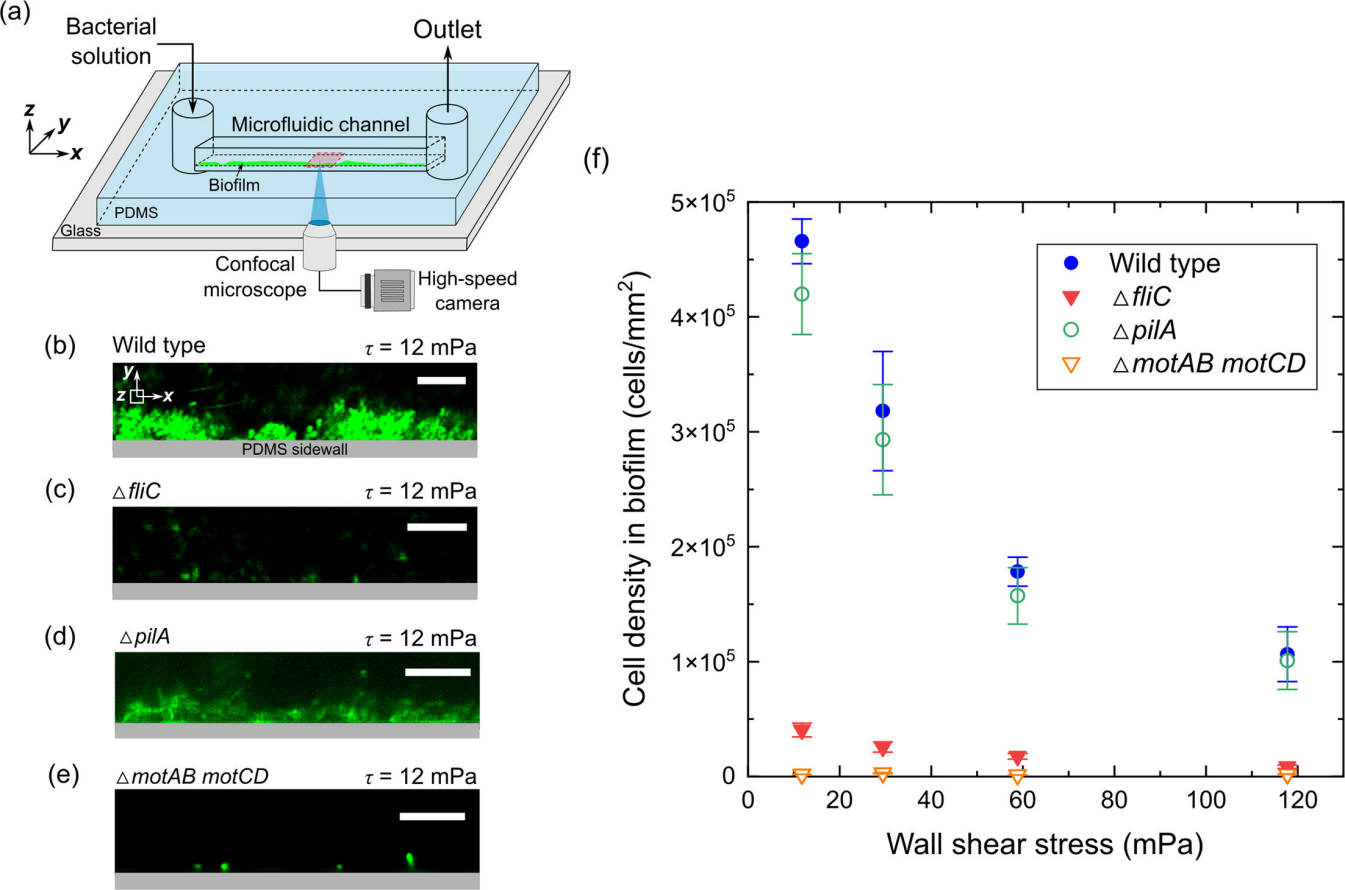
Biofilm formation by motile and non-motile bacterial cells under varying flow conditions. (**a**) Schematic of the experimental setup. (**b**) Fluorescence microscopy image showing biofilms formed by motile *Pseudomonas aeruginosa* (wild-type) cells, labeled with GFP. (**c**) Fluorescence microscopy image showing scattered distribution of non-motile GFP-labeled *P. aeruginosa* (Δ*fliC*) cells. (**d**) Fluorescence microscopy image showing biofilms formed by motile but pilus-deficient (Δ*pilA*) cells. Biofilms were stained with dyes specific to exopolymeric substances. (**e**) Fluorescence microscopy image showing scattered distribution of non-motile (Δ*motAB motCD*) cells stained with SYTO-9. Scale bars represent 10 µm for panels **b–e**. The shear stress is 12 mPa for panels **b–e**. (**f**) Comparison of cell density within biofilms (cells/mm²) among motile (WT and Δ*pilA*) and non-motile (Δ*fliC*, Δ*motAB motCD*) cells under varying shear stress over a 15 hour injection period. Error bars indicate the standard error of the mean from three replicate experiments.

To investigate how cell motility affects *Pseudomonas aeruginosa* biofilm formation, we compared biofilms formed in a microfluidic channel by *P. aeruginosa* wild-type (WT) cells with those formed by two mutant strains: Δ*fliC* (flagellar mutant) and Δ*pilA* (type IV pilus mutant). We visualized biofilm development under a constant wall shear stress ranging from 12 to 120 mPa (Fig. 1a). At low shear stress of 12 mPa, after 15 hour cell injection, biofilms of the wild-type strain were observed on the channel sidewalls, reaching a thickness of approximately 10 µm (Fig. 1b). Fluorescent images of a dye specific to exopolymeric substances confirm that they were produced within these biofilms (Fig. S1). In contrast, the non-motile Δ*fliC* mutant only showed sparse single cells on the sidewalls across all shear stress levels considered, rather than forming structured biofilms (Fig. 1c). In addition to the flagellum-deficient Δ*fliC* mutant, we repeated the experiments using the pilus-lacking Δ*pilA* mutant and observed biofilms similar to those formed by the wild-type strain (Fig. 1d). To confirm the role of pili, we conducted complementation experiments under both low and high shear stress (12 and 120 mPa). The biofilm formation capability of the complemented strain (Δ*pilA pilA*+) was restored to levels comparable to the WT cells under similar flow conditions (Fig. S2). This suggests that pili may not be essential for *P. aeruginosa* biofilm formation in this context, despite their crucial role in twitching movement and surface adherence (23, 25).

To further examine whether flagella contribute to biofilm formation through motility rather than surface adhesion, we tested a non-motile flagellated strain (Δ*motAB motCD*), which retains flagella but lacks the ability to rotate them for swimming. Similar to Δ*fliC*, the Δ*motAB motCD* strain did not form biofilms under any of the tested shear conditions, instead showing only sparse single cells on the channel sidewalls (Fig. 1e). Together, these findings indicate that flagellum-driven cell motility is a critical factor determining the formation of *P. aeruginosa* biofilms.

We further compared the cell density for each mutant under different wall shear stresses (Fig. 1f). For the range of shear stress considered here, 12–120 mPa, motile cells consistently exhibited a larger biomass compared to their non-motile counterparts. At the low shear stress of 12 mPa, the cell density of biofilms formed by motile cells was approximately 10 times higher than that of biofilms formed by non-motile mutants. For the motile strain, as wall shear stress increased, the density of cells on the sidewalls decreased, consistent with a previous study, which showed elevated shear forces inhibit the formation of *Pseudomonas putida* biofilms (38). These findings indicate a complex interplay between cell motility, shear stress, and biofilm formation. A fundamental understanding of how the flagellum-driven motility of individual cells regulates biofilm formation is essential to reveal this complex interaction.

### Motile cells move toward surfaces

**Fig 2:**
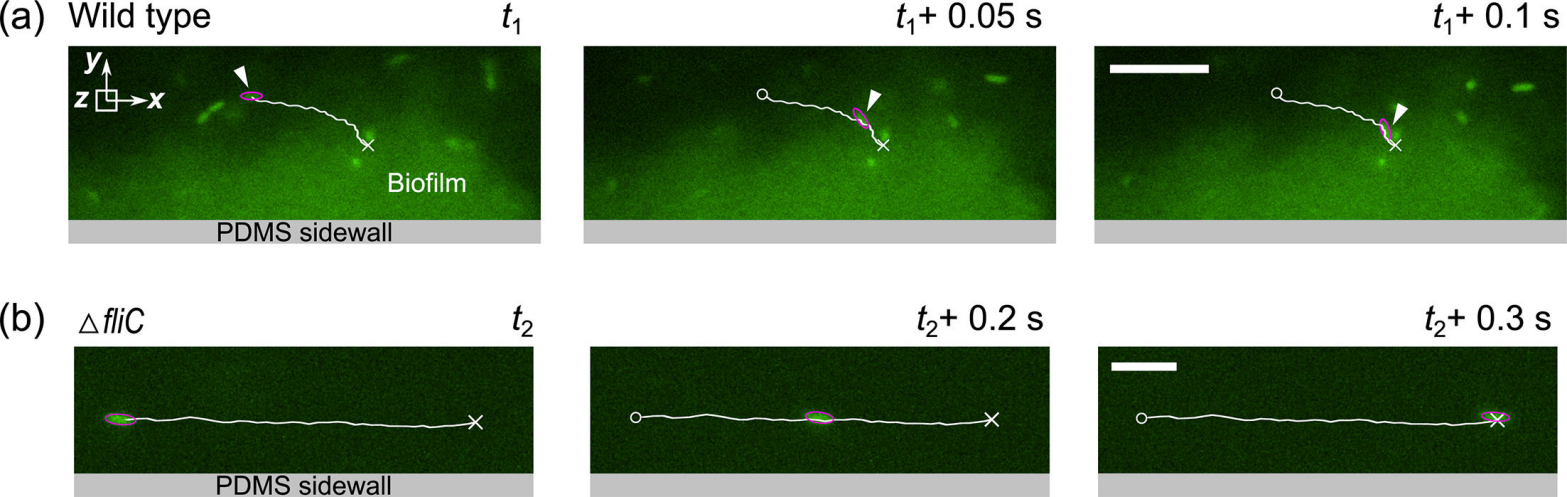
Representative trajectories of motile and non-motile *Pseudomonas aeruginosa* cells near the sidewall. (**a**) Sequential fluorescent microscopy images capture the attachment of motile GFP-labeled cells to the biofilm. The white triangles point to one representative cell that moved toward and got attached to the biofilm (the green blur), with its trajectory shown by a solid line. The circles and crosses denote the start and end points of the trajectory, respectively. The green blur represents the biofilm structure slightly out of the focal plane. Scale bars represent 10 µm. (**b**) Representative sequential fluorescent images showing the trajectory (solid line) of a non-motile (Δ*fliC*) cell flowing along a streamline and not attaching to the sidewall. The circles and crosses denote the start and end points, respectively. Scale bars represent 5 µm. The circles and crosses represent the start and end points of the trajectories, respectively. *y* = 0 represents the PDMS sidewall surface.

To further investigate how flagellum-driven motility facilitates biofilm formation, we tracked the trajectories of motile and non-motile *P. aeruginosa* cells using a high-speed camera. Some typical trajectories of the WT cells and the Δ*fliC* mutant at low shear stress (12 mPa) are shown in Fig. 2a and b, respectively. Under a low shear stress of 12 mPa, the trajectories of the flagellum-lacking Δ*fliC* mutant cells aligned with the fluid flow streamlines (Fig. 2b; Movie S2), resulting in less attachment to the sidewall and limiting biofilm formation. In contrast, many wild-type cells moved toward and subsequently adhered to the sidewalls, forming biofilms (Fig. 2a; Movie S1). The trajectories of pilus-lacking Δ*pilA* cells were similar to the WT cells, further confirming that the wall-directed movement was controlled by flagella. At increased shear stress (120 mPa), the trajectories of both WT and Δ*fliC* cells aligned with the streamlines (Fig. S3), consistent with the observation that no biofilms were developed for both strains at shear stress greater than 120 mPa (Fig. 1e). These findings suggest that while non-motile cells primarily move with the fluid flow, many motile *P. aeruginosa* cells can move toward the sidewall at low shear stress (*τ* < 12 mPa), leading to enhanced biofilm formation shown in Fig. 1.

### Wall-normal angle controls biofilm development

**Fig 3:**
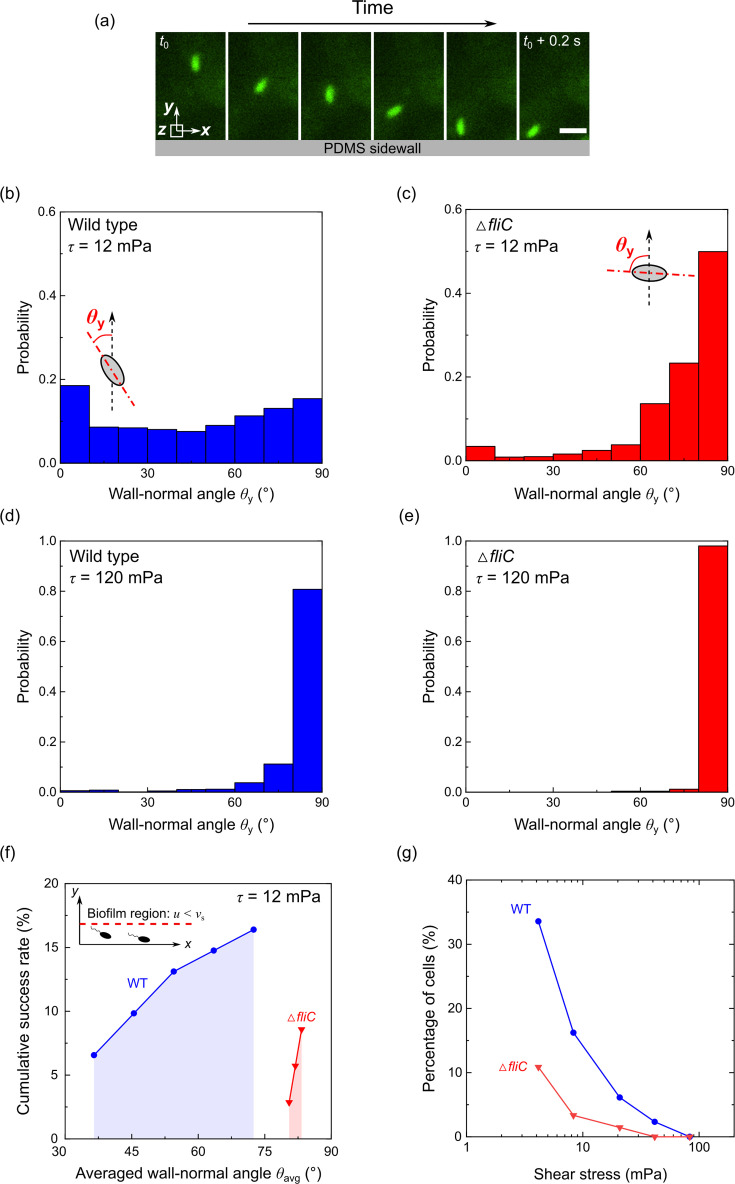
Cell wall-normal angle in response to fluid flow. (**a**) Time-lapsed fluorescence microscopy images demonstrate typical behavior of motile cells moving toward and attaching to the PDMS sidewall under shear stress of 12 mPa during a 0.2 s period. Scale bar represents 5 µm. Panels **b** and **c** illustrate the probability distribution of the wall-normal angle for wild-type and non-motile (Δ*fliC*) cells under the same shear stress of 12 mPa, respectively. Insets show the definition of cell wall-normal angle: the angle between the cell’s axis and the *y*-axis. Panels **d** and **e** show the probability distribution of the wall-normal angle for wild-type and non-motile (Δ*fliC*) cells under the same shear stress of 120 mPa, respectively. Each statistical analysis used around 300 trajectories from three biological replicates. (**f**) The cumulative success rate to reach the biofilm-forming region (defined as regions where the local velocity *u* is less than the cell swimming speed *v*_*s*_) as a function of the average wall-normal angle *θ*_avg_ (averaged for each single trajectory) under shear stress of 12 mPa. The inset depicts the definition of the biofilm-forming region. (**g**) The percentage of cells in the biofilm-forming region as a function of shear stress.

In addition to analyzing cell trajectories, we observed that the motile cells can adjust the orientation of their major axis to approach the sidewalls (Fig. 2a and 3a). Using fast single-cell imaging, we traced the orientation of wild-type and Δ*fliC* cells close to the sidewalls and calculated the wall-normal angle, *θ*_*y*_, defined as the angle between the cell’s major axis and the *y*-axis (Fig. 3b). At lower shear stress (12 mPa), the wall-normal angle of the motile WT cells exhibited a much more uniform distribution with a slight peak at lower angles in the range of 0°–30° (Fig. 3b). In contrast, non-motile (Δ*fliC*) mutant cells predominantly had wall-normal angles between 75° and 90° (Fig. 3c), aligning mainly with the fluid flow direction. These results suggest that many motile cells can actively orient themselves toward the sidewalls, or low-velocity region, rather than following the streamlines. The increased wall-directing orientation of motile cells may be driven by two factors: active movement toward the wall and increased angular diffusivity of the motile cells. We count both factors as active orientation of the motile cells toward the wall (29, 48, 49). At high shear stress (120 mPa), the wall-normal angle of both motile and non-motile cells became comparable, predominantly falling in the range of 75°–90° (Fig. 3d and e). This suggests that the advantage of motility in biofilm formation is not significant under higher fluid shear (Fig. 1f).

Furthermore, we investigated how the success rate of cells reaching the biofilm-forming region varies with the average wall-normal angle (averaged for each single trajectory) for both motile and non-motile cells (Fig. 3f). The biofilm-forming region is defined as regions where the local fluid advection velocity *u* is less than the cell swimming speed *v*_*s*_ (38). We calculated the success rate as the percentage of cells in the biofilm-forming region relative to the total number of cells observed within the region of interest (ROI; see Materials and Methods for details) after the same duration of 3 s. At the shear stress of 12 mPa, 16% of wild-type motile cells reached the biofilm-forming regions, while only 8% of the non-motile Δ*fliC* mutant cells reached such regions. Motility increases the success rate as the motile cells can actively orient themselves toward the sidewalls, leading to higher cell density and biofilm formation near the wall. Most of the motile cells that reached the biofilm-forming regions had wall-normal angles between 30° and 60°. In contrast, non-motile cells mostly had a large wall-normal angle, between 80° and 90°, causing them to follow the streamline and seldom reach the low-velocity region. As shear stress increased to 120 mPa, the percentage of both motile and non-motile cells reaching the biofilm-forming region decreased (Fig. 3g). However, motile cells (WT) maintained an overall higher percentage compared to non-motile cells (Δ*fliC*) across all levels of shear stress. The correlation between the success rate of cells entering the biofilm-forming region and the cell wall-normal angle confirms that cell orientation plays a critical role in facilitating bacterial attachment to walls and subsequent biofilm formation.

### Shear stress controls bacterial cell orientation

**Fig 4:**
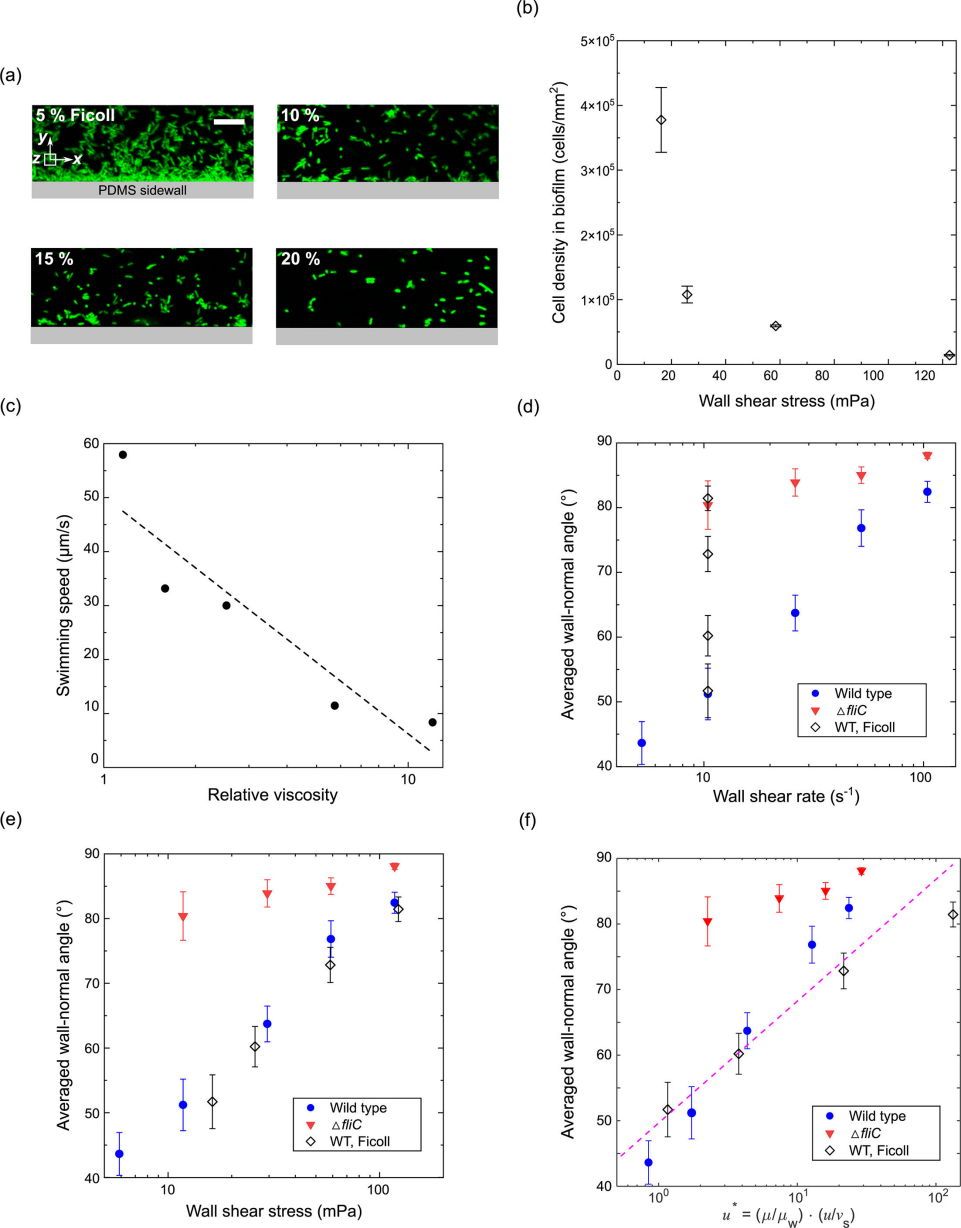
Shear stress-induced cell orientation and enhanced biofilm formation. (**a**) Fluorescence microscopy images of biofilms formed by wild-type *Pseudomonas aeruginosa* at increasing Ficoll concentrations (5%, 10%, 15%, and 20%), which increases viscosity while maintaining constant shear rate. Scale bar: 10 µm. (**b**) Cell density within biofilms (cells/mm²) as a function of wall shear stress for wild-type *P. aeruginosa* under Ficoll-supplemented conditions (open diamonds). (**c**) Relative viscosity as a function of the corresponding bacterial swimming speed measured across Ficoll concentrations. The fitted trend line follows: *y* = − 44log_10_*x* + 50. (**d**) Average wall-normal angle of bacterial cells as a function of wall shear rate. (**e**) Average wall-normal angle of bacterial cells as a function of wall shear stress. (**f**) Wall-normal angle as a function of the non-dimensional relative velocity u^*^. The fitted trend line follows: *y* = 18log_10_*x* + 50.

To determine whether cell orientation and subsequent biofilm formation are regulated by shear stress or shear rate, we systematically varied the fluid viscosity by adding Ficoll at different concentrations (5, 10, 15, and 20 wt.%) to the nutrient solution and repeated the experiments at a fixed shear rate (10 s^−1^). At the same shear rate (10 s^−1^), as the fluid viscosity increases from 1.5 to 11.7 mPa·s, the cell density of biofilms decreases by 90% (Fig. 4a and b), suggesting that biofilm formation is regulated by near-wall shear stress rather than shear rate. To investigate how viscosity affects bacterial motility, we measured the swimming speed of wild-type cells in solutions with varying Ficoll concentrations (Fig. 4c). As shown in Fig. 4e, swimming speed decreases by approximately sevenfold as fluid viscosity increases from 1.1 to 11.7 mPa·s. This reduction in motility suggests that bacterial swimming becomes less effective at higher viscosities.

We then quantified the average wall-normal angles of bacterial cells at each fluid viscosity and plotted these as a function of both wall shear rate and shear stress (Fig. 4d and e). The results show that the orientation angle of motile cells scales with shear stress, not shear rate, confirming that fluid shear forces, rather than velocity gradients alone, control bacterial alignment near surfaces and consequently influence biofilm formation. To further quantify this effect, we introduced a non-dimensional relative velocity: u^*^ = (*μ*/*μ*_*w*_) (*u*/*v*_*s*_), where *μ* is the viscosity, *μ*_*w*_ is the viscosity of water, *u* is the averaged flow velocity, and *v*_*s*_ is the cell swimming speed. When cell orientation is plotted against this non-dimensional parameter (Fig. 4f), data from all conditions collapse onto a single curve, demonstrating that bacterial alignment is governed by the force balance between fluid shear and swimming-driven motility.

### Motility facilitates biofilm formation under competitive conditions

**Fig 5:**
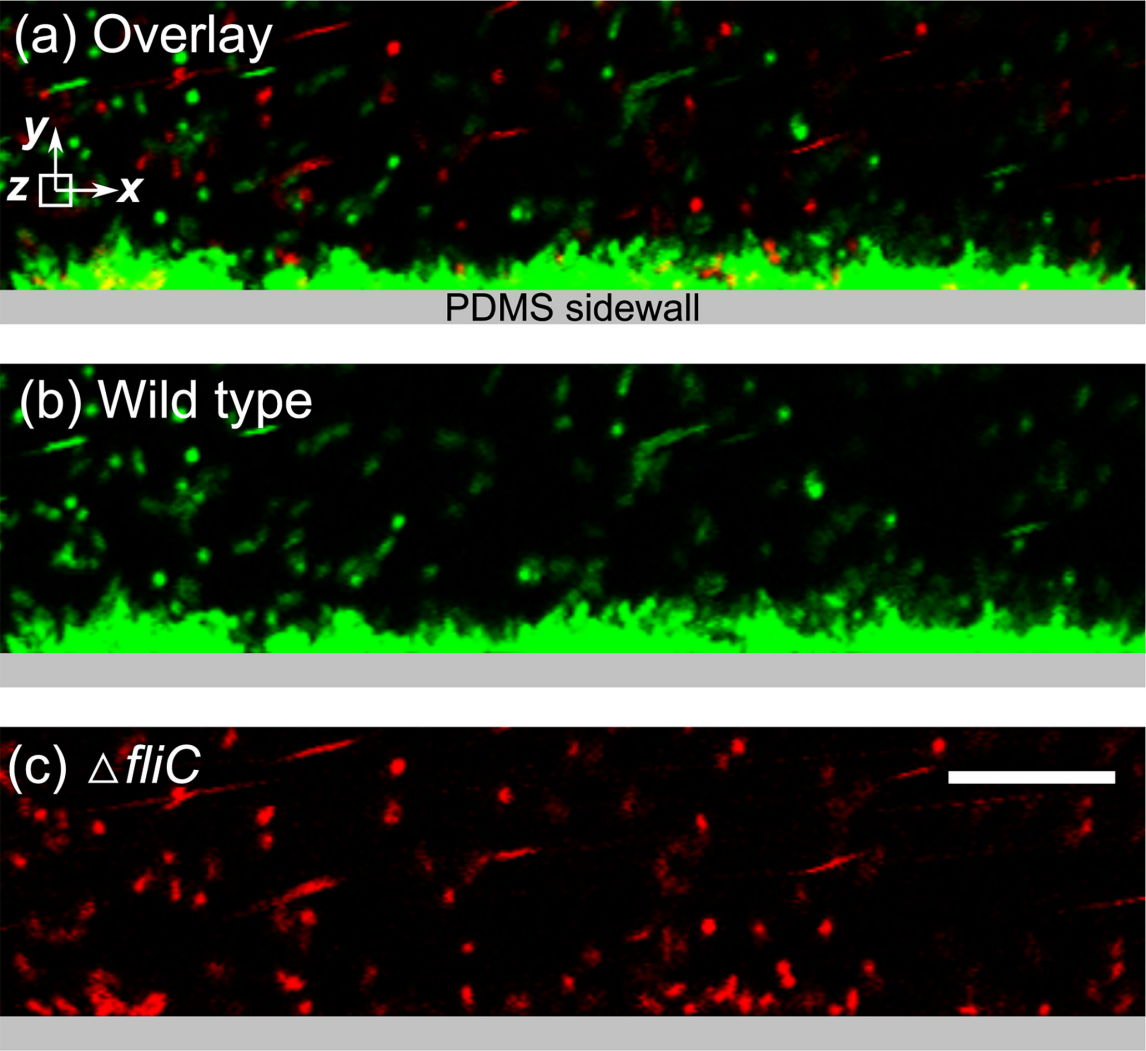
The competitive advantage of motility on biofilm formation. (**a**) Overlap of GFP and mCherry images showing the dominance of motile cells in the biofilm. Yellow color indicates the co-existence of the GFP- and mCherry-labeled cells. The experiment involved injecting GFP-labeled motile and mCherry-labeled non-motile *Pseudomonas aeruginosa* cells into the microfluidic chamber at the same cell density under a shear stress of 12 mPa for a 15 hour injection period. (**b**) Formation of a dense biofilm by GFP-labeled motile cells after a 15 hour experiment. (**c**) Distribution of scattered aggregates of mCherry-labeled non-motile cells (Δ*fliC*) within the microfluidic chamber. Scale bars represent 20 µm.

Furthermore, to understand how motility facilitates biofilm formation under competitive conditions, we injected the *P. aeruginosa* solution containing both motile (wild-type, GFP-labeled) and non-motile (Δ*fliC*, mCherry-labeled) cells at a 1:1 ratio into the microfluidic channel. After 15 hours of injection, we observed that the biofilms formed on the sidewalls were predominantly composed of motile cells (Fig. 5a). Motile cells demonstrated superior ability to colonize the sidewalls of the microfluidic channel, forming dense biofilm structures (Fig. 5b). In contrast, non-motile cells were mostly unable to form substantial biofilms and remained scattered as small aggregates (Fig. 5c). This observation indicates that motility provides a competitive advantage for motile cells to colonize surfaces. This finding can inform strategies to control biofilms in medical, industrial, and environmental contexts.

## DISCUSSION

Previous studies demonstrated that both flagella and type IV pili are critical for biofilm formation under flow environments (22, 27). For example, Klausen et al. (50) showed that both flagella and pili play crucial roles at different stages of biofilm formation under continuous flow conditions, with pili primarily promoting irreversible surface attachment and flagella facilitating initial surface encounter. Similarly, Anyan et al. (51) reported that type IV pili modulate bacterial collective behavior during surface swarming by promoting intercellular interactions. More recently, Palalay et al. (25) showed that shear forces could enhance bacterial adhesion by reducing pilus-mediated detachment, highlighting the complex interaction between type IV pilus activity and surrounding fluid forces. Once cells are attached to a solid surface, type IV pilus-mediated twitching motility enables them to move and migrate upstream (23, 24). In contrast, our study focuses on free-swimming cells in the bulk fluid, where we observe that flagellum-induced active orientation allows cells to navigate toward sidewalls, enhancing surface attachment and subsequent biofilm formation. Similarly, Khong et al. (30) showed that flagellum-driven near-wall swimming behaviors promoted the attachment of *P. aeruginosa* cells to PDMS wall surfaces and the subsequent biofilm growth. Our results further suggest that Δ*fliC* mutants form significantly fewer biofilms and provide a quantitative measurement of the cell orientation. A recent study by Ramachandran et al. (26) found that motile cells tend to accumulate near sidewalls under flow, consistent with our observations. Our work uniquely investigates bacterial swimming motility in the bulk fluid, focusing specifically on how flagellum-driven motility, rather than pilus-mediated interactions, influences the initial approach and attachment to surfaces under flow. Our results reveal that motile cells utilize flagellar motility to reach surfaces by maintaining trajectories favorable for attachment (e.g., smaller wall-normal angles), which enhances initial surface interactions. Moreover, we demonstrate that fluid shear force (e.g., shear stress) plays a crucial role in this process, directly influencing the orientation and surface approach trajectories of swimming cells. In this study, all observations were focused on bacterial interactions with the vertical PDMS sidewalls of the microfluidic device. While surface properties such as hydrophobicity, charge, and stiffness are known to influence bacterial attachment, our aim was to isolate the effects of motility and shear stress on initial attachment dynamics. A comparison between PDMS and other substrates, such as glass, would be valuable in future work to further understand how surface material modulates attachment behavior.

Notably, some studies have reported that *Pseudomonas aeruginosa* mutants deficient in flagella, such as those lacking flagellin (Δ*fliC*), overproduce exopolymeric substances, potentially as a compensatory mechanism for the loss of motility (52). Our results show that all strains, including wild type, Δ*fliC*, and Δ*pilA* mutants, were able to produce detectable levels of exopolymeric substances (Fig. S1). However, the Δ*fliC* mutant formed significantly less biofilms compared to the wild-type strain. These results suggest that motility plays a more critical role in the initial stages of biofilm formation under the conditions of our experiments. Previous research has also demonstrated that extracellular DNA (eDNA) influences biofilm formation by facilitating cell aggregation and adhesion to surfaces (37). Further studies are needed to elucidate how different motility mechanisms, in combination with factors like eDNA, collectively contribute to biofilm formation across diverse microbial species.

Additionally, we examined whether the reason motile cells move toward the sidewalls is due to chemotaxis, the movement of cells in response to a chemical concentration gradient (53, 54). In the present study, the sidewalls of the chamber are made from gas-permeable PDMS, making it possible to generate an oxygen gradient between the center of the channel and the sidewalls. To investigate whether it is the oxygen gradient that drives the cell toward the wall, we conducted a series of experiments using a gas-impermeable plastic chip (cyclic olefin copolymer). We observed that motile *P. aeruginosa* cells exhibited consistent swimming behavior (calculated average wall-normal angle around 50°) toward the sidewalls in both the gas-permeable and gas-impermeable chambers (Movie S3). Furthermore, the wall-directing motion of motile cells occurred at the beginning of the experiments, when there were no visible cells on the sidewalls, indicating that the wall-directing motion of motile cells was not driven by extracellular signaling materials produced by the cells.

Our results indicate that cell orientation is governed by shear stress rather than shear rate, indicating that bacterial behavior is force-dependent. Based on this observation, we propose that motile cells orient toward the wall due to shear stress gradients, a mechanism previously described in other studies (26, 55, 56). The shear stress differences on the cell head and its tails can generate a torque to facilitate the cell’s navigation toward the low-velocity region near the wall. At low shear stresses (*τ* < 12 mPa), bacterial motility can overcome the torque on the cell body, allowing cells to migrate into biofilm-forming regions. However, at high shear stresses (*τ* > 120 mPa), bacterial motility becomes insufficient to overcome the strong flow, reducing the likelihood of cells accumulating near the wall and forming biofilms. These results further confirm that the interplay between bacterial cell motility and fluid shear plays a crucial role in regulating biofilm formation. Furthermore, we observed no biofilm growth under no-flow conditions (Fig. S4), likely due to the absence of nutrient and oxygen supply without flow.

Overall, our study demonstrates the role of motility and flow in biofilm formation and provides a quantitative framework to predict these effects. Our findings have implications for managing biofilm-related issues in medical, industrial, and environmental contexts. The role of motility in enhancing biofilm formation suggests that targeting bacterial motility could be an effective approach for controlling biofilm development. The fundamental understanding of the control of motility and fluid flow on biofilm development could contribute to the design of biofilm-resistant surfaces and the development of antimicrobial strategies.

## MATERIALS AND METHODS

### Bacterial culture

The details of the *Pseudomonas aeruginosa* PA14 strains we used here are described in Table S1. Bacterial solutions were prepared following the steps below. First, *P. aeruginosa* cells were cultured from a frozen stock and incubated in Luria Broth solution overnight (~16 h) at 37°C with 200 rpm shaking. Second, the bacterial solutions were transferred to a modified M9 solution with a fully characterized chemical composition (38, 57) [supplemented with 0.03 M (NH_4_)_6_(Mo_7_)_24_, 4 M H_3_BO_3_, 0.3 M CoCl_2_, 0.1 M CuSO_4_, 0.8 M MnCl_2_, 0.1 M ZnSO_4_, and 0.1 M FeSO_4_]. Specifically, we centrifuged 5 mL of bacterial cultures at 3,000 × *g* for 10 min and then removed the supernatant. The bacterial deposit was then diluted with M9 medium solution to an OD_600_ of about 0.5. D-glucose at 1 wt.% concentration was added to the M9 medium as a carbon source. For the Ficoll experiments, we added 5, 10, 15, and 20 wt.% Ficoll 400 (Sigma) to the bacterial suspension to systematically increase the viscosity. The relative viscosity of each solution was measured using a digital rotational viscometer (CGOLDENWALL NDJ-5S, China).

### Biofilm development experiments

A schematic diagram of the experimental platform is shown in Fig. 1a. The system consists of a microfluidic chip, a Confocal Laser Scanning Microscope (C2 plus, Nikon, Japan), a high-speed camera (ORCA-FLASH 4.0, Hamamatsu Photonics, Japan), and a programmable syringe pump (PHD Ultra, Harvard Apparatus). Soft lithography was used to fabricate polydimethylsiloxane (PDMS) microfluidic chips with the assistance of the University of Minnesota Nano Center. The straight channels utilized in this study have a height of 60 µm and a width of 400 µm. The channel measures 5 mm in length from inlet to outlet. The PDMS channel is bonded to a #1.5 cover glass (Electron Microscopy Sciences, USA) using a corona plasma treater (Electro-Technic Products, USA) for a 30 second surface treatment. All the experiments were conducted under room temperature (~22°C) to minimize cell growth, as this study primarily focuses on investigating the swimming and attachment behaviors of cells. Additionally, the growth curves of motile and non-motile cells showed no significant difference (Fig. S5), allowing us to exclude the difference in cell growth as a variable affecting biofilm growth.

Biofilm development experiments were conducted following the steps described below (Fig. 1a). First, 2 mL of the nutrient solution (abiotic M9 solution containing 1 wt.% D-glucose) was injected into the microfluidics to displace the air in the channel. Then, we continuously injected *Pseudomonas aeruginosa* bacterial solution (OD_600_ = 0.51 ± 0.02) into the microfluidic channel at five different flow rates (*Q* = 0.5, 1, 2.5, 5, and 10 µL/min) for 15 hours. The shear rate ranged from 5 to 100 s^−1^. The corresponding calculated wall shear stress (*τ*) at the sidewall surfaces was 6, 12, 30, 60, and 120 mPa, respectively (*τ =* 6*ηQ/hw*^2^*,* where *η =* 1.13 mPa·s is fluid dynamic viscosity, *Q* is flow rate, *h*  =  60 µm is channel depth, and *w* = 400 µm is channel width). The Reynolds number in our experiments ranged from 0.04 to 0.72, corresponding to laminar flow conditions. This range is comparable to flow regimes found in natural and biomedical environments, including groundwater flow through porous media, fluid flow in medical devices, and blood flow in small vessels (58–60). To ensure accurate comparisons, we injected either wild-type or mutant cells into the microfluidic channel under identical conditions. To determine if motile cells are directed toward the walls due to chemotaxis, we conducted biofilm development experiments in a gas-impermeable channel following the same procedure. The channel, made of a cyclic olefin copolymer, measures 200 µm in width, 100 µm in depth, and 18 mm in length.

### Confocal microscopy visualization

During the experiments, we recorded biofilm development over time using a Nikon C2+ Confocal Laser Scanning Microscope with 0.3 μm horizontal resolution at a quarter-depth, close to the bottom glass surface. The wavelength of the laser was 488 nm. The objective magnification was 20×. During the experiments, the images were scanned at 30 minute intervals and saved on an HP-Z4-G4 workstation. Bacterial trajectories and orientations were captured using epifluorescence microscopy (60× objective magnification) and a high-speed CMOS camera (ORCA-FLASH 4.0, Hamamatsu Photonics, Japan) with a speed of 200 fps.

### Exopolymeric substance staining in microfluidic channels

To determine whether *P. aeruginosa* biofilms secrete exopolymeric substances, we stained the biofilms with exopolymeric substance-specific dyes after the 15 hour cell injection experiments (9, 38). A microfluidic channel with three inlets was used to stain exopolymeric substances. We first injected nutrient solution through the first inlet to flush the channel and remove bubbles. Afterward, we introduced *Pseudomonas aeruginosa* cell culture through the second inlet. At the end of the experiment, a staining solution specific to exopolymeric substances was injected through the third inlet for 10 minutes to stain the biofilms. The staining solution consisted of 5 µM of SYTO-9, a green fluorescent nucleic acid stain (Thermo Fisher Scientific), as well as 20 µg/mL fluorescein isothiocyanate (FITC) conjugated Concanavalin A (ConA) from *Canavalia ensiformis* (Sigma), and 20 µg/mL of FITC-conjugated wheat lectin (WGA) from *Triticum vulgaris* (Sigma). The SYTO-9 binds to extracellular DNA. ConA binds to α-D-mannose, α-D-glucose, and alginate. WGA binds to N-acetyl-D-glucosamine and N-acetylneuraminic acid.

### Image analysis

To quantify biofilm growth, we calculated the cell density in biofilms, defined as the number of cells near the sidewall per square millimeter (cells/mm^2^) (38). In all cases, biofilm cell density was quantified from mono-channel images. First, we selected a region of interest close to the biofilm boundaries and calculated its area using Image-J. The width of the ROI was set at 50 pixels away from the sidewall, and its area was the same for all cases. Second, we identified the area occupied by the biofilms in the ROI and calculated the biofilm-covered area in MATLAB. Then, we calculated the cell numbers by dividing the area of the biofilms by the area of one cell. To estimate the cell density, defined as the number of cells per surface area (cells/mm^2^), we divided the number of cells by the area of the ROI. The trajectories and cell wall-normal angle were calculated using the TrackMate function in ImageJ (61). Specifically, we employed the Thresholding detector and Nearest-neighbor tracker to detect cell movements. Cell trajectories were reconstructed in MATLAB. The cell wall-normal angle was derived from the ellipse angle in ImageJ following a coordinate transformation. Additionally, these angles were subjected to manual verification to ensure accuracy.

### Statistical analysis

For each experimental condition, three biological replicates were conducted. The results are shown as the mean ± standard error. The mean value was calculated from three replicates. The error bars indicate the standard error of three replicates.

## Data Availability

All trajectory data, MATLAB codes for image processing, and microscopic images are available in the Data Repository for the University of Minnesota (DRUM): https://doi.org/10.13020/tq68-ww73.

## SUPPLEMENTAL MATERIAL

Supplemental material aem.00821-25-s0001.docx Figures S1 to S6, Table S1, and legends for Movies S1 to S3.

Movie S1 aem.00821-25-s0002.avi Time-lapse video showing motile wild-type *P. aeruginosa* cells changing their orientation to reach and attach to the biofilms.

Movie S2 aem.00821-25-s0003.avi Time-lapse video showing non-motile *P. aeruginosa* cells (Δ*fliC*) predominantly moving aligned with the fluid flow streamlines.

Movie S3 aem.00821-25-s0004.avi Time-lapse video showing motile wild-type *P. aeruginosa* cells exhibiting similar behaviors: reorienting and adhering to sidewalls in a non-gas-permeable environment.

ASM does not own the copyrights to Supplemental Material that may be linked to, or accessed through, an article. The authors have granted ASM a non-exclusive, world-wide license to publish the Supplemental Material files. Please contact the corresponding author directly for reuse.
