# Supplementary material for: Flagellum-driven motility enhances Pseudomonas aeruginosa biofilm formation by altering cell orientation
Source: Appl Environ Microbiol. 2025 Jul 3;91(7):e00821-25. doi: 10.1128/aem.00821-25 (PMC12285247; doi:10.1128/aem.00821-25)
Supplement: Supplemental material — Figures S1 to S6, Table S1, and legends for Movies S1 to S3. [file aem.00821-25-s0001.docx]

**Supporting Information**

**Flagellar-Driven Motility Enhances *Pseudomonas aeruginosa* Biofilm Formation by Altering Cell Orientation**

Guanju Wei ^a, b^, Jessica-Jae S. Palalay ^c^, Joseph E. Sanfilippo ^c^, Judy Q. Yang ^a, b*^

^a^ Saint Anthony Falls Laboratory, University of Minnesota, Minneapolis, MN 55414, USA

^b^ Department of Civil, Environmental, and Geo-Engineering, University of Minnesota, Minneapolis, MN 55455, USA

^c^ Department of Biochemistry, University of Illinois at Urbana-Champaign, Urbana, IL 61801, USA

**Corresponding Author:**

^*^Judy Q. Yang ([judyyang@umn.edu](mailto:judyyang@umn.edu))

**This includes:**

Figure S1 to S6

Table S1

Legends for Movies S1 to S3

**Other supporting materials for this manuscript include:**

Movies S1 to S3

**
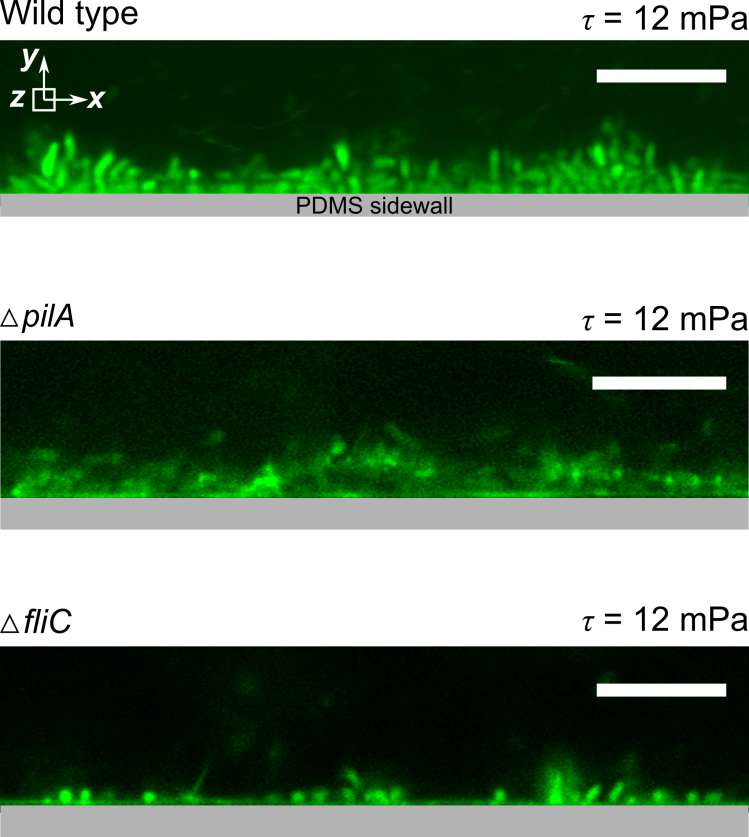
**

**Fig. S1:** Fluorescence microscopy images showing exopolymeric substances staining of wild-type, Δ*pilA* and Δ*fliC* strains after a 15-hour growth period under identical shear stress conditions of 12 mPa. Biofilms and aggregates were stained with exopolymeric substance-specific dyes (see Methods for details). Scale bar represents 10 μm.


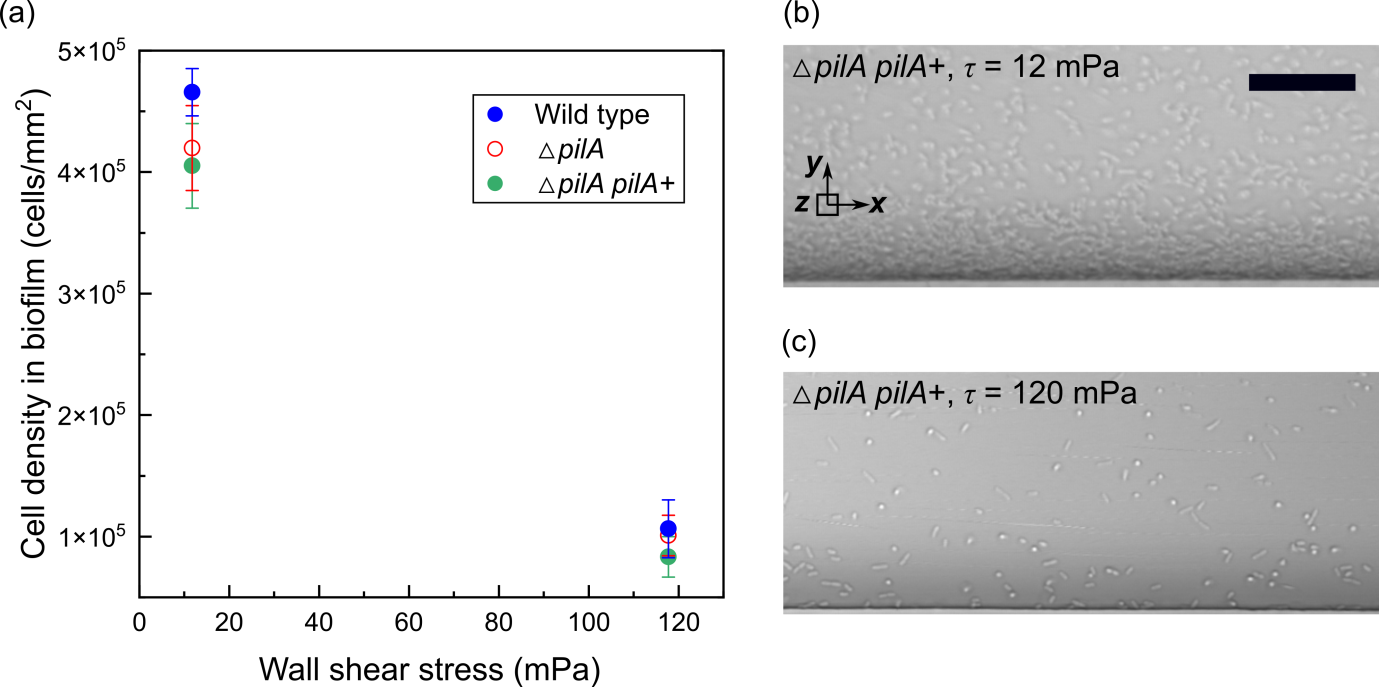


**Fig. S2:** Complementation experiment of Δ*pilA* *pilA*+. **(a)** Comparison of cell density within biofilms (cells/mm²) among *Pseudomonas aeruginosa* wild-type, Δ*pilA* and Δ*pilA* *pilA*+ cells. **(b**) Microscopy image showing biofilms formed by Δ*pilA* *pilA*+ cells under low shear stress of 12 mPa. **(c)** Microscopy image showing only scattered distribution of Δ*pilA* *pilA*+ cells under high shear stress of 120 mPa. Scale bars represent 20 μm for both (b) and (c). The mean value was calculated from three replicates. The error bars indicate the standard error of three replicates.

**
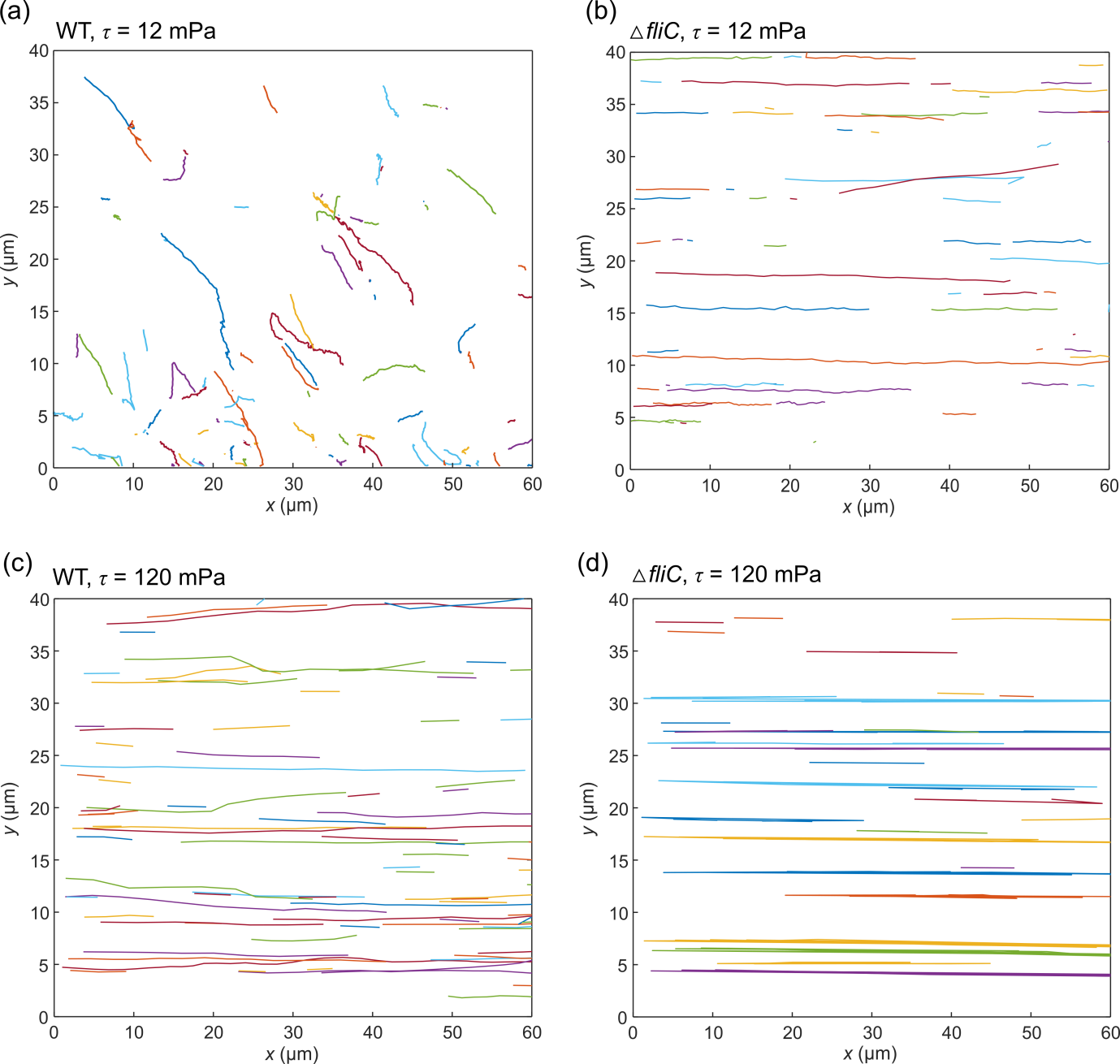
**

**Fig. S3:** All Trajectories in the ROI of *Pseudomonas aeruginosa* wild-type and Δ*fliC* cells under **(a, b)** low shear stress of 12 mPa and **(c, d)** high shear stress of 120 mPa. Different colors distinguish individual trajectories for visualization.


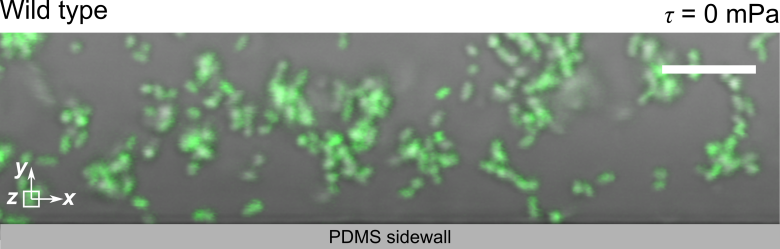


**Fig. S4:** Under no-flow conditions, no biofilms were observed on the sidewalls after a 15-hour period. The green colors indicate only cell aggregates formed in the bulk fluid. Scale bar represents 10 μm.

**
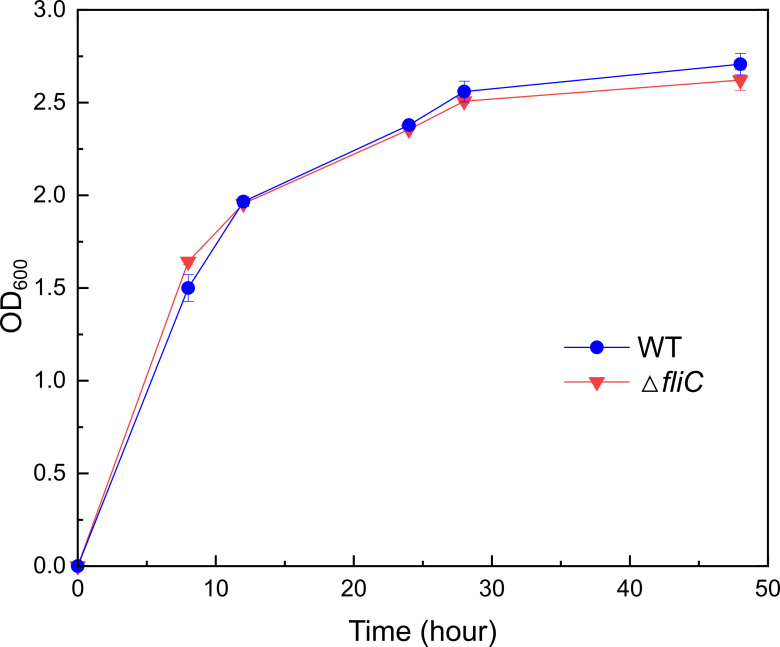
**

**Fig. S5:** Growth curves of *Pseudomonas aeruginosa* in Luria Broth (LB) solution. The error bars represent standard error for three replicates.


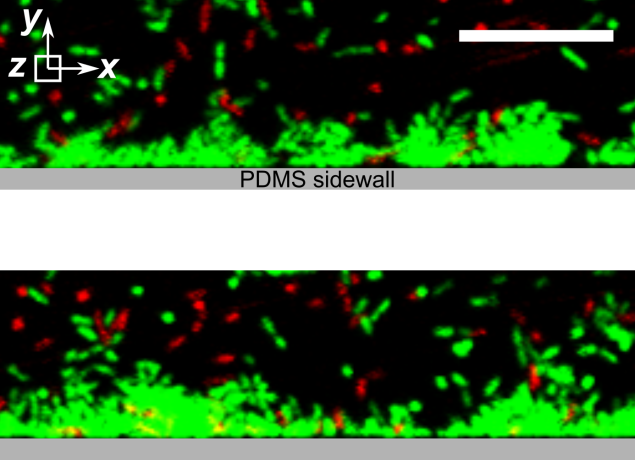


**Fig. S6:** Replicates demonstrating the competitive advantage of motile cells in biofilm formation. GFP-labeled motile and mCherry-labeled non-motile *Pseudomonas aeruginosa* cells were co-injected into a microfluidic chamber at equal initial densities under a shear stress of 12 mPa for 15 hours. These images are representative of multiple experimental replicates. Scale bars represent 20 μm.

**Table S1.** *P. aeruginosa* strains used in this study

| ***P. aeruginosa* strain** | **Description** | **Source** |
| --- | --- | --- |
| JS1 | Wild-type PA14; clinical isolate from burn wound | [1] |
| JS11 | *attTn7::[plac-GFP aacC1::FRT]* | [2] |
| JS27 | Δ*pilA::FRT* | [3] |
| JS28 | Δ*fliC::FRT* | [3] |
| JS192 | Δ*fliC::FRT*; expressing mCherry | This paper |
| JS204 | Δ*fliC::FRT*; expressing GFP | This paper |
| JS213 | Δ*motCD::FRT ΔmotAB::FRT* | This paper |
| JS218 | *Tn7::PpilA-pilA::FRT* | This paper |

[1]. L Rahme, E Stevens, S Wolfort, J Shao, R Tompkins, and F Ausubel, *Science* **268**(5219),1899-1902 (1995).

[2]. A Siryaporn, S Kuchma, G O'Toole, and Z Gitai, *PNA*S **111**, 16860-16865 (2014).

[3]. J Sanfilippo, A Lorestani, M Koch, B Bratton, A Siryaporn, H Stone, and Z Gitai, *Nat. Microbiol.* **4**, 1274-1281 (2019).

**Movie S1.** Time-lapse video showing motile wilt-type *P. aeruginosa* cells changing their orientation to reach and attach to the biofilms (highlighted in the white box). Flow direction is from left to right with a shear stress of 12 mPa.

**Movie S2.** Time-lapse video showing non-motile *P. aeruginosa* cells (Δ*fliC*) predominantly moving aligned with the fluid flow streamlines. Flow direction is from left to right with a shear stress of 12 mPa.

**Movie S3.** Time-lapse video showing motile wild-type *P. aeruginosa* cells exhibiting similar behaviors: reorienting and adhering to sidewalls in a non-gas-permeable environment (highlighted in the white box). Flow direction is from left to right with a shear stress of 12 mPa.
